# What matters to children with lower limb deformities: an international qualitative study guiding the development of a new patient-reported outcome measure

**DOI:** 10.1186/s41687-021-00299-w

**Published:** 2021-04-01

**Authors:** Harpreet Chhina, Anne F. Klassen, Jacek A. Kopec, John Oliffe, Christopher Iobst, Noemi Dahan-Oliel, Aditya Aggarwal, Tim Nunn, Anthony P. Cooper

**Affiliations:** 1 Department of Experimental Medicine, Faculty of Medicine, University of British Columbia, Vancouver, Canada; 2 Department of Orthopaedics, BC Children’s Hospital, 1D 18, Orthopaedics Research Office, 4480 Oak Street, Vancouver, BC V6H 3V4 Canada; 3 Department of Pediatrics, McMaster University, Hamilton, ON Canada; 4 Department of Surgery, McMaster University, Hamilton, ON Canada; 5 Department of Health Research Methods, Evidence, and Impact, McMaster University, Hamilton, ON Canada; 6 School of Population and Public Health, University of British Columbia, Arthritis Research Canada, Vancouver, BC Canada; 7 School of Nursing, University of British Columbia, Vancouver, BC Canada; 8 Department of Orthopaedic Surgery, The Ohio State University, College of Medicine, Nationwide Children’s Hospital, Columbus, OH USA; 9 Shriners Hospitals for Children, School of Physical and Occupational Therapy, McGill University, Montreal, Quebec Canada; 10 Department of Orthopaedic Surgery, Post Graduate Institute of Medical Education and Research, Chandigarh, India; 11 CURE Ethiopia Children’s Hospital, Addis Ababa, Ethiopia; 12 Department of Orthopaedics, Faculty of Medicine, University of British Columbia, Vancouver, Canada

**Keywords:** Patient- reported outcome instruments, Health- related quality of life, Children, Lower limb deformities, Qualitative interviews, International study

## Abstract

**Background:**

Lower limb deformities include conditions such as leg length discrepancy, lower limb deficiency and associated angular and rotational deformities of the hips, knees, ankles and feet. Children with lower limb deformities often have physical limitations due to gait irregularities and pain. The differences in the appearance and function of their lower limbs can discourage participation in social, recreational and leisure activities, which may result in behavioural, emotional, psychological and social adjustment problems. The health-related quality of life (HRQL) of these children is often impacted due to the factors discussed above, as well as by the complex surgical procedures. Surgical treatment options for limb deformities in children vary from limb lengthening and reconstruction to amputation. The lack of evidence demonstrating superiority of either treatment options and their effect on HRQL limits the ability of healthcare providers to counsel families on the best evidence-based treatment option for them.

This manuscript describes the international qualitative study which guided the development of a new patient-reported outcome measure (PROM). Individual semi-structured face-to-face interviews with children with lower limb deformities and their parents were conducted at five sites: Canada (2 sites), Ethiopia, India and the USA.

**Results:**

Seventy-nine interviews were conducted at five international sites. Five main themes emerged from the qualitative interviews and formed the basis of the conceptual framework. These themes were: 1) appearance, 2) physical health, 3) psychological health 4) school and 5) social health.

**Conclusions:**

Lower limb deformities have a substantial impact on the HRQL of children. The concepts of interest identified in our study were similar across children from all countries. The conceptual framework guided the development of outcome scales specific to these patients.

The information about the impact of various treatment options on the HRQL of children with lower limb deformities, collected using this new PROM, could be used to inform parents and children about outcomes (physical, social, psychological) associated with specific treatment options. This information could supplement other objective outcome information (e.g., complication rates, how the leg will look, etc.) to help families to come to a more informed decision on a child’s course of treatment.

**Supplementary Information:**

The online version contains supplementary material available at 10.1186/s41687-021-00299-w.

## Introduction

Lower limb deformities include conditions such as leg length discrepancy, lower limb deficiency and associated angular and rotational deformities of the hips, knees, ankles and feet. Lower limb deformities can be separated into two broad categories: 1) congenital deformities, which may result from defects in fetal development, and 2) acquired deformities, which arise from trauma, infections, tumours and other medical conditions [1]. Epidemiological data vary among individual deformities. For example, in a retrospective study from France, leg length discrepancy of greater than 2 cm was found in at least 1 in every 1000 people [2]. In the USA, the overall incidence of limb reduction defects is estimated to be 2 per 10,000 live births [3]. The case rate of limb deficiency defects in Canada (excluding Quebec) in 2014 was 3.74 per 10,000 births [4]. Fibular hemimelia a congenital deficiency of the long bones has an estimated incidence between 7.4 and 20 per 1 million live births [5–8]. The incidence of tibial hemimelia is reported to be one per million live births [9, 10]. The incidence of congenital femoral deficiency is 1 in 52,029 [8]. However, there is lack of up to date data on epidemiology of these congenital conditions and lower limb deformities.

Children with lower limb deformities often have physical limitations due to gait irregularities and pain. These differences in the appearance and function of their lower limbs can discourage participation in social, recreational and leisure activities, which may result in behavioural, emotional, psychological and social adjustment problems [11–15]. The health-related quality of life (HRQL) of these children is often impacted due to the anatomic and functional factors discussed above, as well as by the complex surgical procedures for their treatments [16–19].

Treatment options depend on the type of limb deformity, as well as the clinician’s skills and institutional resources available at the treating centre. Non-surgical treatment options include shoe-lifts, braces and step-in prostheses. Surgical treatment options for limb deformities in children vary from limb lengthening and reconstruction to amputation. Choice of treatment is usually dependent on a number of factors including clinical presentation [20], the desires of the patients and parents, the predicted HRQL, cultural influences, cosmetic preference and socio-economic status of individual families [13, 14]. Socio-economic status has been known to be the fundamental cause of health disparities between high-income, lower-middle and low-income countries. Hence, the availability and access to treatments vary considerably between these countries. Furthermore, the high-income countries are using more sophisticated surgical treatment options such internal lengthening devices and latest hexapod external fixators with the ability to correct deformities more precisely, whereas majority of the low-income and lower-middle income countries are still using traditional external fixators when available. Overall, there is less specialist surgical workforce in low- income and lower-middle income countries [21].

Each treatment option has advantages and disadvantages. Amputation is a non-reversible surgical procedure followed by fitting of a prosthesis. Amputation is associated with potential stump overgrowth and pain. Prosthetic limbs require periodic replacement due to patient growth, breakage or fitting problems and lack normal sensation and proprioception [22]. The life-time cost of prosthesis replacement can be substantially higher than the repeat surgical procedures for limb lengthening [23].

Reconstruction by lengthening corrects limb length discrepancy and deformity by utilizing distraction osteogenesis [24]. This approach is more involved and may require multiple procedures. An external or internal fixation device is applied to the leg and remains in place for months. The child experiences multiple hospital visits and extensive physiotherapy and rehabilitation. This treatment is psychologically stressful due to its duration, impact on schooling and the potentially high rate of complications [16, 18, 25, 26].

There is insufficient evidence in the literature to support one treatment option over the other. Some studies show successful outcomes with amputations [22, 27–31], while others have also reported similarly successful outcomes with lengthening and reconstruction [32–36]. Some comparative effectiveness studies have found no difference when comparing amputation and reconstruction [37, 38].

The lack of evidence demonstrating superiority of either treatment options and their effect on HRQL limits the ability of healthcare providers to counsel families on the best evidence-based treatment option for them. Consequently, there is a need for a rigorously designed patient-reported outcome (PRO) instrument to collect data from patients with lower limb deformities. These data could be used to inform parents and children about outcomes (physical, social, psychological) associated with these procedures. This information could supplement other objective outcome information (e.g., complication rates, how the leg will look, etc.) to help families to come to a more informed decision on a child’s course of treatment [39].

Our systematic review (SR) identified the lack of a validated and psychometrically tested patient-reported outcome measure (PROM) for children with lower limb deformities [40]. To fill this gap, our team is developing a new PROM called LIMB-Q Kids [41]. A rigorous qualitative phase is crucial to the development of a new PROM [42]. Qualitative interviews can provide a deeper understanding of what is important to a patient and establish the content validity [43, 44]. In this report, we describe the international qualitative study which guided the development of LIMB-Q Kids. Our aim was to identify concepts of interest important to children with lower limb deformities and using this knowledge to develop a conceptual framework of HRQL for these children.

## Methods

Individual semi-structured face-to-face interviews with children with lower limb deformities and their parents were conducted at five international sites: Canada (2 sites), Ethiopia, India and the USA. Ethics approval was obtained at the primary study site (CW15–0215 / H15–00514) and each of the participating sites. A written informed consent was obtained from the parents and assent was obtained from the children.

### Sampling

Purposive sampling was used to recruit a maximum variation sample of children with lower limb deformities that included patients who varied by type of lower limb deformities, treatment type, stage of treatment, age, gender and country of residence.

### Inclusion criteria

Children aged 8 to 18 years with a confirmed diagnosis of a lower limb deformity were eligible. Parents of these eligible patients were also invited to participate in individual interviews.

### Exclusion criteria

Patients with lower limb deformities along with other medical conditions which may have a confounding effect on their HRQL and conditions such as cognitive or developmental delay affecting their ability to communicate were excluded from this study. Isolated deformities of hip and foot were excluded.

### Study setting, recruitment and data collection

Recruitment took place at hospitals in Canada, the USA (High-income countries), India (Lower-middle income country) and Ethiopia (Low-income country). The two sites from Canada and 1 site from the USA were tertiary care hospitals providing specialised orthopaedic care to children across their respective provinces. The site from India was a leading tertiary care hospital providing care to patients from several states in India. The site from Ethiopia was a pediatric orthopaedic teaching hospital that provided care to physically disabled children. Our two centres in Ethiopia and India primarily catered to patients with low socio-economic status with limited access to medical care.

Recruitment at these sites was done by their respective research teams in consultation with the primary study site (senior author’s site). An interview guide was prepared in English (attached in appendix) and translated into French, Amharic, Hindi and Punjabi. The interview guide was based on the preliminary conceptual framework derived from our SR [40]. Feedback from the orthopaedic surgeon (AC) and an expert in PROM development (AK) also informed the interview guide. The interview guide was modified during the process to gain understanding of emerging themes.

All English interviews in Canada and the USA, and Hindi and Punjabi interviews in India were conducted by the first author (HC). The first author is a native speaker of both Hindi and Punjabi languages and is also fluent in English. Interviews at the French speaking Canadian site and Amharic speaking site in Ethiopia were conducted by their local research assistants who were trained by the first author. Both the researchers from the French speaking Canadian site and Amharic speaking site in Ethiopia were native speakers of French and Amharic respectively. They were both fluent in English aswell. Interviews were conducted until data saturation was reached i.e. no new information or thematic variation emerged in the subsequent interviews [45, 46].

### Data analysis

All interviews were recorded and transcribed verbatim. Hindi and Punjabi interviews were transcribed and translated into English by the first author (HC). French interviews were translated and transcribed by the same researcher from the French speaking site in Canada who conducted the interviews. Amharic interviews were translated into English and transcribed by the research assistant from the Amharic speaking site who also performed the interviews. A pragmatic approach to translation was used in this study. Triangulation was achieved by interviewing the parents or caregivers of the children as a second source of data [47, 48].

The purpose of conducting this qualitative study was to identify prevailing concepts representative of the international sample as a means to developing items for a new patient-reported outcome measure. While analysing the interviews, variables such as age at the time of interview, country of residence, and child or parent interview were included to see if the concepts were relevant across country, age and parent versus child.

This study adopted an interpretive description (ID) approach [49]. ID is a non-categorical qualitative approach which aims at developing knowledge for the clinical context of applied health disciplines [50]. Transcripts were analysed using a line-by-line coding approach to label and identify recurrent themes and patterns. Coding involved an inductive (from the interviews) and deductive process (preliminary conceptual framework from our SR). Constant comparison was used to compare interview data and categorise the concepts of interest (COI) into major and minor themes [51]. The results of this analysis were used to refine the preliminary conceptual framework from our SR [40].

Rigor: We used Guba and Lincoln’s factors to assess four criteria of rigor as suggested by Sandelowski [52, 53]: truth value, applicability, consistency and neutrality. Truth value of a qualitative study is tested by the credibility of the results. Our study findings are supported by relevant quotations from the participants which support the representativeness of those findings. We also used triangulation as a way of ensuring credibility of our results. Triangulation involves collecting data from multiple sources/multiple points of view in an attempt to address the subjectivity in qualitative work and as a way of validating the findings [54]. We achieved triangulation in our study by interviewing the parents/caregivers of these children separately as a second source of data. According to Guba and Lincoln, the applicability of the results of a qualitative study is determined in terms of fittingness [53]. Fittingness refers to the ability of the research findings to fit the data from which they were derived. Quotations from the study participants demonstrated the fit between the data and study findings. The consistency of the qualitative findings is evaluated by their auditability [53]. A description of the coding process and ongoing discussion with the research team members contributed towards the auditability. Peer debriefing, which involved discussing the ongoing analysis with the research team, was also conducted on a regular basis to verify the study findings [51]. To further support auditability, close contact was maintained with the study team members with expertise in qualitative research and PROM development. We used confirmability as the criterion for neutrality, which means that we were reflexive about the research process and the findings [53, 55]. Reflexivity involves awareness of researchers’ biases and beliefs that can influence decisions during the data collection and analysis, including categories not ultimately borne out by the data [56]. The first author also kept a journal to signal thought processes and interpretations, in accounting for how individual patient or parent stories were selected as representative and thematic. The authors involved in developing the coding schedule were non-clinician researchers. Individual investigators from the participating sites were clinicians who were directly involved in the clinical care of children with lower limb deformities. These investigators were involved in the preparation of the manuscript, including a detailed review of the qualitative analysis. The involvement of multiple perspectives and discussions to reach a consensus reduced individual biases.

## Results

**Fig. 1:**
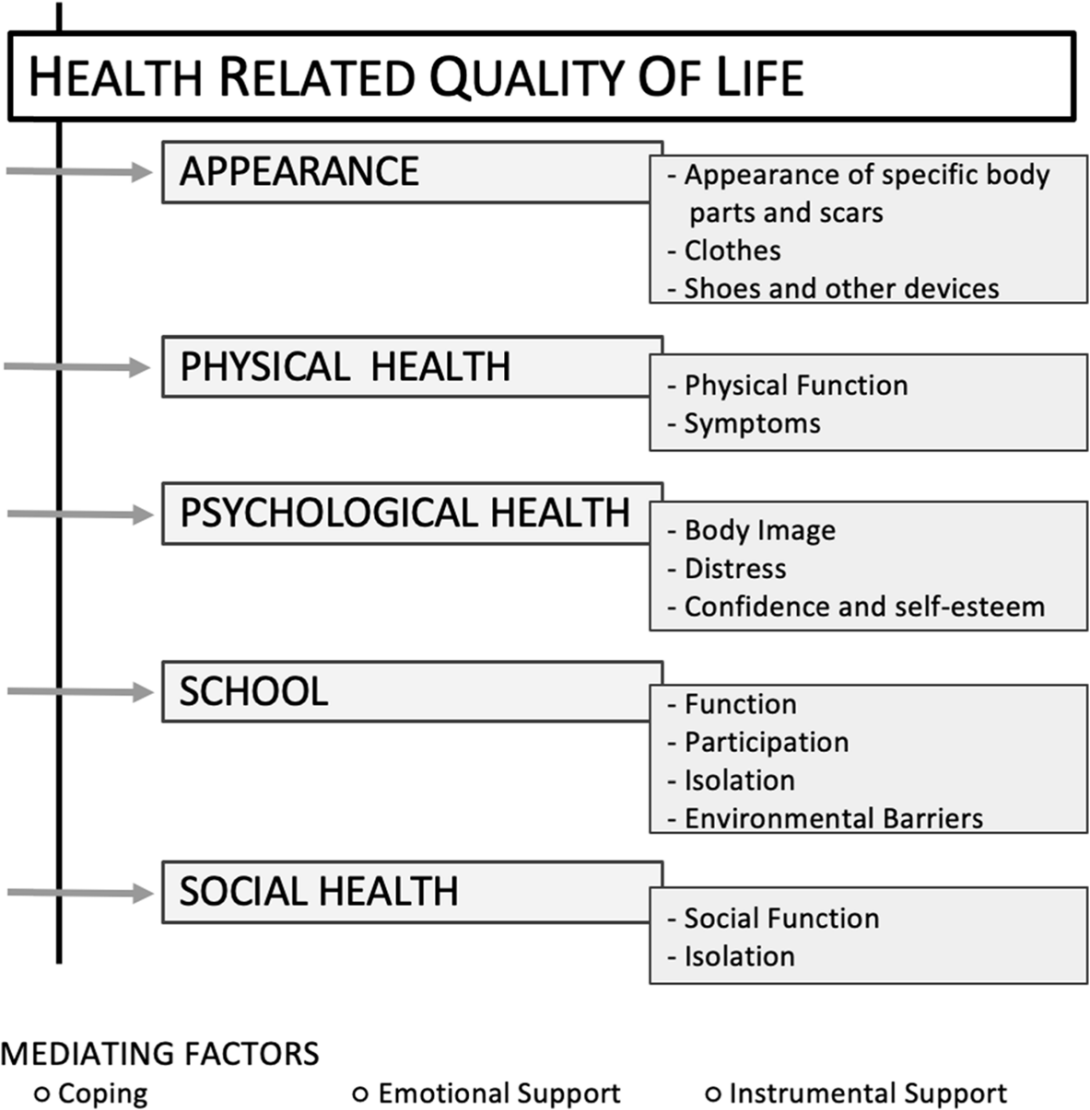
Conceptual Framework

Seventy-nine interviews (39 children and 40 parents) were conducted with children with lower limb deformities and their parents from four countries (Canada, Ethiopia, India and the USA) (Table 1). Interviews were conducted at individual sites between years 2015–2018. Out of the 39 interviewed children, 2 had amputation, 7 had no treatment at the time of the interview, 20 were in active treatment phase (external fixators, internal lengthening nails, osteotomy) and 10 had some treatment done in the past (external fixators, internal lengthening nails). A list of lower limb deformities of the interview participants is included in the appendix (Table 2). Five main themes emerged from the qualitative interviews and formed the basis of the conceptual framework (Fig. 1). These five themes were: 1) appearance, 2) physical health, 3) psychological health 4) school and 5) social health. These themes formed the basis of development of outcome scales for the LIMB-Q Kids. Other factors such as coping, emotional and instrumental support were identified to be the mediating factors influencing HRQL. Below we describe each theme and provide illustrative quotes (Additional file 2: Table S1) from interviewed children (labelled as ‘C’) and their parents (labelled as ‘P’). Though we conducted individual interviews with children and their parents separately, no new themes emerged from the parent interviews only.

1. **Appearance:** Leg appearance was an important theme for the interviewed children.
  - **Appearance of specific body parts and scars**: Appearance was discussed in terms of the leg overall, specific parts including hips, knees, feet and surgical scars (Quotes 1–11). The children talked specifically about the shape, symmetry, size and overall appearance of their legs (Quotes 1–4).
  - **Clothing:** Children also discussed how their leg looked when wearing different types of clothing and foot-wear (Quotes 12–16). Children talked about the type and fit of clothes and overall appearance with certain clothes (Quotes 13–17). They discussed how they were not able to wear different types of clothes as they would like. Some children talked about not being able to wear shorts or short skirts and they wore long pants to hide their leg. They also described having to wear specific types of clothes when they had an external fixator device (Quotes 13, 14). Adolescent children were also concerned about the appearance of their legs in photographs (Quote 19).
  - **Shoes and other devices:** Children talked about the appearance when wearing treatment related devices such as splints, crutches, external fixator devices, braces, prosthesis and shoe-lifts (Quote 17–18).
2. **Physical Health:** Children talked about their limitations with physical function and their symptoms.
  - **Physical function:** Under physical function, the children discussed their limitations with mobility, balance, sports and recreational activities, activities of daily living (ADL) and their adaptations for specific activities. Most children were unable to walk or run long distances without discomfort or pain (Quotes 20–23). The mobility limitations were either due to their limb deformity or due to the shoe-lifts or external fixator devices.
    Children also mentioned losing balance, feeling uneven and falling (Quote 26–27). They described limitations in the type of sports they could participate in due to their limb deformity and treatment related devices such as external fixators and shoe- lifts (Quote 30–32). Some children mentioned choosing alternate sports or adapting themselves accordingly (Quotes 28–29). Most children were able to perform ADLs without any limitations even when they were wearing prosthesis. However, some children mentioned having problems with ADLs (putting on or taking off clothes etc.) while they were in the external fixator devices (Quote 24).
  - **Symptoms:** Children also talked about the symptoms they experienced due to their lower limb deformity. Symptoms included pain, feeling tired and limping (Quotes 33–36). Pain involved the leg as well as other parts of the body. Pain was described in terms of its frequency and severity (Quotes 37–41). Pain was reported during the time period when they had the external fixators on. They also discussed interference of pain during walking and sleeping at night (Quotes 33–34, 41).
3. **Psychological Health:** Lower limb deformities impacted the psychological health of children. Children shared their concerns about their body image, talked about the distress due to their leg related problems, how their leg related problems impacted their confidence and self-esteem and how they developed coping strategies. Some children indicated how their leg problems had a positive impact on them.
  - **Body image:** Children with leg deformities perceived their body image diversely. Some children with congenital deformities perceived themselves as being ‘normal’ since they were born with the lower limb deformity. At the same time children who acquired the deformity due to trauma, infection, tumours or other medical conditions later on in their life perceived themselves as not being 'normal' and experienced challenges adapting physically and psychologically (Quotes 42- 44).
    Children described a range of strategies to conceal their leg with deformity or their leg with the external fixator device. Some avoided wearing certain types of clothes while others would not go out with clothes that revealed their deformity (Quotes 45–46). Some children with congenital deformities walked with an altered gait to conceal their limp (Quote 46). Some children felt ashamed or embarrassed about their body image with their limb deformity (Quote 45). Children described that they felt self-conscious due to their leg deformity, limping, external fixator devices or the shoe-raises they had to wear (Quotes 47–50).
  - **Distress:** Most children had experienced leg related-distress to some extent either due to their physical limitations, appearance related issues due to the deformity, external fixator devices or shoe raises, complex surgical procedures and limitations in social participation. Their distress was expressed in various emotions such as being worried, feeling down, crying, feeling irritated, and angry (Quotes 57–68). Children were worried about their surgeries and how their leg problems would affect them in the future. (Quote 53–56). They were worried about limping, other people staring, not being able to walk and the leg length difference happening again after they had received the treatment. While some adolescents were also worried about the kind of jobs they can do in future with their leg problem, younger children worried about their leg or foot hurting while playing. Children also felt being down, sad, upset and discouraged at times due to the challenges they were facing either due to the deformity itself or due to the treatment procedures such as having the external fixator device on for months (Quotes 57–68).
  - **Confidence and Self-esteem:** Children talked about how their leg problems and external fixator devices affected their confidence and self-esteem (Quotes 69–75). Some of them didn’t feel comfortable getting their pictures taken while others felt inferior to other kids (Quote 75).

**Table 1:** Participant Demographics

	Canada - 1	Canada - 2	USA	India	Ethiopia	Total
Interviews (parent and patient) Total	16	10	21	20	12	79
Interviews Patients	7	5	11	10	6	39
Congenital Lower Limb Deformity	6	5	11	7	5	34
Acquired Lower Limb Deformity	1	0	0	3	1	5
Average Age (in years for patients)	13.7	11	12.3	13.7	13	12.9
Age Range (in years for patients)	11 to 18	8 to 18	9 to 16	11 to 18	10 to 17	8 to 18
Gender						
Female	2	2	7	2	2	15
Male	5	3	4	8	4	24
Interview in English						
Yes	16	0	21	0	0	37
No	0	10	0	20	12	42

**Table 2:** List of deformities included in the study sample

• Acquired genu valgum post treatment of Ewing's Sarcoma, leg length discrepancy due to growth disturbance due to 8 plates for treatment of genu valgum	
• Acquired leg length discrepancy secondary to a tibial fracture	
• Congenital A/K amputation	
• Congenital femoral deficiency	
• Fibular hemimelia	
• Congenital pseudarthrosis of tibia	
• Genu valgum	
• Infantile Blount’s Disease	
• Infection induced proximal tibial growth plate disturbance, proximal tibia vara, shortening and recurvatum deformities	
• Leg length discrepancy secondary to congenital posterior medial tibial bowing	
• Leg length discrepancy, Perthes	
• Leg length discrepancy, osteogenesis imperfecta	
• Leg length discrepancy secondary to left hemi-hypertrophy	
• Leg length discrepancy, post traumatic avascular necrosis of hip	
• Leg length discrepancy, arm deformities secondary to meningococcemia	
• Leg length discrepancy acquired due to post traumatic malunion of femur	
• Leg length discrepancy secondary to septic hip, osteomyelitis, avascular necrosis of hip	
• Leg length discrepancy	
• Leg length discrepancy, osteoporosis	
• Leg length discrepancy, osteogenesis imperfecta, osteoporosis	
• Leg length discrepancy, neuroblastoma at birth	
• Leg length discrepancy, congenital patella dislocation	
• Tibial deficiency (Gallop-wolfgang complex)	
• Right femoral hypoplasia	
• Right hemihypertrophy, KT syndrome	
• Right external tibial torsion, miserable mal-alignment syndrome, osteochondritis dissecans	
• Rickets, severe bilateral leg deformities	
• Proximal femoral focal deficiency	
• Post traumatic partial distal tibial physeal closure with severe deformity of the ankle

**4) School**: Most children talked about the impact of having a limb deformity on their school life. They discussed their limited participation in activities at school, having to miss school, the isolation they felt at school, emotional and instrumental support from their friends and teachers and some school-based environmental barriers.

- **Function**: Children mentioned having to miss school because of their frequent appointments with the doctors, physiotherapists and occupational therapists and recovery period after surgeries. While some children were ok going to school with the external fixator devices on others were scared to go to school (Quotes 83–92). Most children interviewed from Canada and the USA were able to go to school while some children from India and Ethiopia had to miss a lot of school or were not able to go to school at all during the treatment phase. They either had to travel to different cities for treatment or had physical limitations getting to school with their deformity because they couldn’t afford the treatment (Quotes 87–92). Some children missed school due to pain and other complications of their surgical procedures (Quotes 91–92).
- **Participation**: Children talked about their limited participation at school in academic, sports and recreational activities. Most participants did not describe any impact on their academic performance due to their limb deformity. However, a few children from India and Ethiopia mentioned missing an entire year of school due to treatment (Quotes 93–95). Participation in sports and other activities at school was impacted for most children. Some children found alternate activities that were physically less demanding to accommodate their lower limb deformity or while they were in the external fixator devices (Quotes 93–95).
- **Isolation:** Children felt isolated and left out at times due to being teased by their peers, being asked about their legs all the time, having to tell other people about their leg, shoe-lifts and external fixator devices (Quotes 104–108).
- **Environmental barriers:** Children mentioned having problems at school due to the environmental barriers such as taking the stairs with their external fixator devices and problems when they were in wheelchair (Quotes 102–103).

5) ** Social Health**: All children interviewed were school age so most of their social life was around friends at school or school related social activities. However, some children did talk about the impact of their deformity on the social life outside of school.
  - **Social Function:** Children talked about how their social function was affected in terms of making new friends, going out with friends and family (Quotes 109–110). Their limited physical function and concerns about their appearance directly impacted their social function in terms of being unable to participate in social activities with friends and family (Quotes 111–112). Adolescent children mentioned how they would want to post pictures on social media. Some adolescent children also shared their difficulties in finding jobs especially the children with prosthesis (Quotes 112, 116–117).
  - **Isolation:** Children also felt socially isolated and left out when their friends and other people noticed their limping, stared at their leg and asked them about their leg. Some experienced teasing from peers. They felt lonely and isolated when they couldn’t go out and play with their friends and siblings (Quotes 125–136).

### Mediating factors

The mediating factors, as identified based on the qualitative analysis, were the factors impacted by the deformity that influenced the children’s perceptions of their HRQL. Coping, emotional and instrumental support were the three mediating factors found in this study.

### Coping

It was also evident from the interviews that some children with lower limb deformities had developed their own coping strategies to deal with their challenges (Quotes 76–82). One child mentioned keeping a journal to document their feelings while another accepted their deformity philosophically, suggesting that everything happens for a reason. Another child also mentioned feeling better thinking that their problem was not as bad as some other people's. Some children coped with their scars by considering them to be reminders of what they have been through. There was a positive impact of their lower limb deformity seen on some children. They felt stronger and more motivated to do certain activities which they couldn’t do during their treatment especially involving the external fixator device. Strong religious beliefs and acceptance of their deformity as god’s will were also seen in interviewed participants from Ethiopia.

### Emotional and instrumental support

Children talked about the emotional support they had at school from their friends and teachers (Quotes 96–98). They had instrumental support from their friends and teachers at school such as needing help carrying a backpack (Quote 99–101). Children also talked about the emotional and instrumental support from friends, people and family (Quotes 118–124).

## Discussion

The aim of this study was to identify domains of HRQL important to children with lower limb deformities and to develop a conceptual framework of HRQL concepts that are important to these children. This framework guided the development of items and scales for an internationally applicable PROM for this patient population.

The preliminary conceptual framework developed based on our systematic review identified three overarching health concepts including physical, psychological and social health [40]. These concepts identified in our systematic review were also consistent with studies looking at the impact of tumours in the lower limb on the HRQL [57–63]. A systematic review by Bekkring et al. summarizes 12 studies, looking at the HRQL, functional ability and physical activity in children and adults with bone cancers of the lower limb [64]. While the overarching health concepts identified in our international qualitative study remain the same as identified in our systematic review, we found certain themes in our qualitative study that were not emphasized in the literature but were important to the children with lower limb deformities. For example, appearance of the leg was an under-represented theme in the literature that was found to be an important theme in the final conceptual framework derived through the interviews. Children talked about the appearance of legs, knees, feet and hips. This is now reflected in the conceptual framework emphasizing the importance of appearance for these children. Table 3 (in Appendix) shows how each theme was endorsed in the interviews.

Similarly, school was a major theme based on the qualitative interviews. Given that our target patient group is between the ages of 8 to18 years when children typically go to school, it is not surprising that most children talked about school-based challenges. Our findings of lower participation in physical activities at school and missed days of school are in line with the similar findings from previous studies [65, 66].

Our findings of concealment by wearing specific clothes or avoiding more revealing clothes, and hiding their affected leg were similar to strategies for hiding upper limb deficiencies [67, 68]. Children discussed several issues with the external fixator devices. These issues were found to be temporary and only encountered during the time they used the external fixators. However, it is common for children to require treatment with an external fixator multiple times over the course of their childhood with the duration of use as long as nine months each time.

 The strength of this study is the heterogeneity of the sample for interviews, in terms of the age, gender, type of deformity, type of treatment, stage of treatment, country of residence and socio-economic status. This diverse sample enabled us to identify a range of concepts important to an eclectic international population of children with lower limb deformities, increasing the generalizability of the PROM. The overall thematic findings resonated with and fairly represented the entire sample included in this study.

With the aim of understanding what’s important to children with lower limb deformities, we also interviewed the parents of these children. A recent review indicating the current recommendations for proxy reports suggests that observable concepts are often reliable while the unobservable concepts such as emotional well-being and social functioning are found to be unreliable when measured using the proxy reports [69]. Similar discrepancies between child and parent proxy reports have been reported in a study looking at HRQL in children with congenital lower limb deficiencies using generic HRQL instruments [14]. The analysis of parent data in our study did not find any new themes other than those found from the interviews with children. This is also supportive of the previous literature suggesting that by the age of 8 years children can reliably report on their well-being, psychological health and health promoting behaviours [70–72].

### Limitations

We acknowledge the potential for sampling bias given the centres selected for qualitative interviews. Since our centres in Ethiopia and India primarily serve patients with low socio-economic status some of these children might have reported substantially higher impact of their limb deformity on the HRQL. However, we did not collect any information on the socio-economic status of the participating children. This conjecture is based on author’s knowledge of the individual participating sites and may be reflective of their bias toward this population. For this study, we were only able to interview children from four countries. The number of children with amputations included in this qualitative study was small. Hence, we might have missed some concepts relevant to children with amputations. 

We do recognise the importance of establishing trustworthiness of translating and transcribing verbatim qualitative interview data. The methodological challenges in cross-language qualitative research are well recognised [73]. A strength of our study is that members of the study team who translated the interview guide, performed interviews in local languages, translated and transcribed them into English were the native speakers of the local language and were fluent in English aswell. A pragmatic approach to translation was used in this study.

While there are limitations of not using a certified translator at this step, using researchers from the study team, with bilingual skills, as translators offers an unique advantage of not bringing in an ‘outsider’ to conduct, translate and transcribe these qualitative interviews. The interviewers had significant background knowledge about their individual sites, the orthopaedic conditions, were well aware of the study objectives and were able to probe efficiently using conceptually equivalent terms as needed.

## Conclusion and future work

Our study describes the impact of lower limb deformities on the HRQL of children. This impact is further influenced by health inequities including low socio-economic status and access to healthcare in lower-middle- and low-income countries. Nonetheless, the COI identified were similar across children from all countries. This detailed knowledge gained from interviewing children with lower limb deformities has provided us with first-hand information about what matters most to these children. This framework guided the development of outcome scales specific to these patients. An exhaustive list of items was generated based on the qualitative interviews. Physical health, psychological health, social health, school and appearance were the areas most important to children with lower limb deformities. Cognitive debriefing interviews with children with lower limb deformities are in progress at the participating sites along with the feedback from clinicians at each site. Next steps include translation and cultural adaptation of the LIMB-Q Kids and an international field test study.

### Supplementary Information

**Additional file 1.**

**Additional file 2.**

## Data Availability

As the raw data for this study is qualitative interviews which can pose a risk to the confidentiality of the participants; this data will not be available openly. However, authors will try their best to answer any questions from the readers and for any future studies in this area.

## Appendix

**Table 3:** Saturation Table Showing the Number of Interviews Endorsing Each Theme and Sub-Theme

Interviews (***N*** = 79)																			
		Canada Site - 1			USA				Canada Site - 2		Total (%)	India				Ethiopia			Total (%)
**Themes**	**Sub-themes**	1–5	6–10	11–16	17–21	22–26	27–31	32–37	38–42	43–47		48–52	53–57	58–62	63–67	68–72	73–77	78–79	
Appearance	Appearance of body parts and scars	4	4	3	1	3	2	3	4	3	27 (47)	4	3	5	3	3	1	0	19 (59)
Appearance	Clothes	1	1	2	2	0	0	1	0	2	9 (17)	3	1	4	3	3	1	1	16 (50)
Appearance	Shoes and other devices	1	2	2	1	0	0	3	0	0	9 (17)	0	1	3	1	1	0	0	6 (19)
Physical Health	Physical Function	4	2	3	1	2	2	4	3	2	23 (40)	2	3	2	3	3	4	2	19 (59)
Physical Health	Symptoms	1	3	2	1	1	1	1	2	3	15 (30)	1	3	2	2	2	2	2	14 (44)
School	Function	1	1	3	0	0	0	2	0	0	7 (13)	1	1	1	3	3	4	1	14 (44)
School	Participation	1	3	2	1	0	0	2	1	1	11 (21)	2	2	2	2	2	1	1	12 (38)
School	Isolation	3	2	1	0	0	0	1	0	0	7 (9)	2	0	2	0	0	0	0	4 (13)
School	Environmental Barriers	0	0	0	0	0	0	0	0	0	0	0	0	0	1	1	0	0	2 (6)
Social	Social Function	2	1	1	1	0	1	1	3	2	12 (21)	3	2	2	2	2	3	2	16 (50)
Social	Isolation	2	2	3	1	2	1	2	1	1	15 (28)	3	4	3	4	4	3	1	22 (69)
Psychological Health	Body Image	2	4	3	1	1	1	2	3	0	17 (32)	2	2	3	3	3	2	1	16 (50)
Psychological Health	Distress	2	2	4	2	0	2	2	3	2	19 (36)	3	2	2	3	3	3	1	17 (53)
Psychological Health	Confidence and self-esteem	3	0	2	1	0	0	1	3	0	10 (15)	1	0	0	3	3	1	1	9 (28)
Mediating Factors																			
Coping		0	1	3	0	0	0	0	1	0	5 (11)	1	0	0	0	0	0	0	1 (3)
Emotional Support		2	2	2	0	0	0	0	2	0	8 (17)	2	0	2	1	3	1	0	9 (28)
Instrumental Support		2	1	2	0	0	0	1	2	1	9 (19)	1	1	2	1	1	1	1	8 (25)

## Abbreviations

HRQL: Health- related quality of Life
PRO: Patient- reported outcome
SR: Systematic review
COI: Concepts of interest
PROM: Patient- reported outcome measure
