# Supplementary material for: What matters to children with lower limb deformities: an international qualitative study guiding the development of a new patient-reported outcome measure
Source: J Patient Rep Outcomes. 2021 Apr 1;5:30. doi: 10.1186/s41687-021-00299-w (PMC8017030; doi:10.1186/s41687-021-00299-w)
Supplement: Supplementary file 1 — Additional file 1. [file 41687_2021_299_MOESM1_ESM.docx]

Development of a Quality of life Instrument for Children with Lower Limb Deformities - Qualitative Interviews

Child Interview Script

This script will serve as a guide for interviewing children ages 8-18 years. Some questions might not be relevant for age group 8-13 years such as question 8 and 13.

Introduction

Introduce self, Explain study; Read information sheet together

Read through consent form together and sign if not done already.

"As we go along, please ask questions at any time. Do you have any questions before we begin?"

Questions/probes

1. Please tell me a little bit about yourself and your family

2. Tell me about your problems with your leg.

Probe: What are your biggest challenges with your leg?

3. Can you tell me about the treatment you received for your leg problem?

4. Was your treatment an overall positive or negative experience?

5. What were the best things about the treatment?

6. What were the biggest challenges with the treatment for you?

7. What were your expectations from the treatment?

8. What were you hoping to get out of your treatment?

9. How did the type of treatments offered meet or differ from your expectations?

Probe: what do you expect/hope your leg to look like after treatment?

10. How do you think your leg problems have affected you?

11. How do you think your challenges with your leg have affected you?

Probe: Is there anything about your leg that bothers you?

12. How do you think has your leg problems affected how you look?

13. What do you currently like/dislike about the appearance of your leg?

14. What are your experiences of finding clothes appropriate for your leg problems?

15. What are your experiences of finding shoes/footwear appropriate for your leg problems?

16. Have you ever had problems finding appropriate clothes?

Probe: wearing shorts, skirts. Dresses?

17. Have you ever had problems finding appropriate footwear/shoes?

18. Is there anything that you would like to change about how your leg looks?

19. How important is your leg's appearance to you?

20. Do you worry about the future impact of the appearance of your leg? If so, how?

Probe for descriptive detail

21. How does the appearance of your leg make you feel?

Probe: happy, sad, anxious, worried, frustrated, self-conscious.

22. How do you feel about scars due to the frames/surgeries for your leg problems?

23. Did you ever have to wear shoe lifts/splints?

24. How do you feel about wearing them? Different footwear/shoe modifications?

Probe: self conscious about shoe raise?

25. How does the function of your leg make you feel?

Probe: happy, sad, anxious, worried, frustrated, self-conscious

26. How does your leg problem affect your function in day-to-day activities?

27. Do you worry about limping?

28. Do you worry about abnormal walking?

29. Do you worry about running?

30. What happens when someone new meets you for the first time?

31. How do you accomplish tasks compared to other kids of your age?

32. How do you feel your leg problems will affect your future function in day-to-day activities?

33. How has your leg problems affected how you feel?

34. Do you feel that someone with a lower limb problem has a better, worse, or same life as other people?

35. How has your leg problems affected your behavior?

36. How has your leg problems affected your self-esteem?

37. How has your leg problems affected your confidence?

38. How has your leg problems affected your school?

Probe: Have you missed a lot of school due to surgeries, appointments?

Any issues with like concentrating or your attention or memory at school?

39. How does your friends react towards your leg problems

Probe: For adolescents above 15 yrs only perhaps? Having a leg problem and being able to have intimate relationships?

Have you had any particular negative experiences with guys/girls saying things to you?

40. How does your family react towards your leg problems?

41. Probe: Supportive? How did they support?

42. How would you describe the attitudes of your friends and community?

Probe: Supportive or not; how did they support you?

Anything in particular they did to accommodate you

What support services help - and why?

If you were to kinda go out and just like be walking about, meeting people, um, have you felt like people have treated you the same, like strangers or have you felt quite judged and treated differently?

Have you used any other support services or anything or found comfort in any other, like engaging with people with leg problems?

43. Are there any activities/situations you avoid because of your leg problems?

44. What are your most challenging day to day activities with your leg problems?

45. Do you feel like having a leg problem has made your life better or worse or about the same as other people?

46. Is there anything else you would like to talk about your leg problems that we haven't covered so far?

47. Can you please tell me anything that bothers you about your leg, even if its minor and unimportant?

Are you interested in talking to us again for the next step in this study?

**Parent Interview Script**

This interview script is supposed to serve as a guide for interviewing families of children with limb deformities. Some of the question might not need to be asked as they would have been discussed by the family during the discussion.

Introduction

Introduce self, Explain study; Read information sheet together

Read through consent form together and sign if not done already.

"As we go along, please ask questions at any time. Do you have any questions before we begin?"

Background

1. Please tell me a little bit about yourself and your family

Lower Limb Specific Questions

2. Tell me about your experience of having a child with lower limb problems.

3. How would you describe your experience? Positive or negative

Probe: why positive or negative? What made it positive or negative? How can we make it positive?

4. How do you perceive your child's physical appearance due to lower limb problems?

5. How does your child perceive his/her physical appearance due to lower limb problems?

6. What do you think your child currently like/dislike about the appearance of his leg?

7. What do you think your child currently like/dislike about the function of his leg?

8. Is there anything that you would like to change about how your child's leg looks?

9. How important is your child's leg's appearance to you?

10. Do you worry about the future impact of the appearance of your child's leg? If so, how? Probe for descriptive detail

11. How does the appearance of your child's leg make you feel?

Probe: happy, sad, anxious, worried, frustrated, self-conscious.

12. Do you worry about the future impact of the function of your child's leg? If so, how? Probe for descriptive detail

13. How do you feel about scars due to the frames/surgeries for your child's leg problems?

14. Did you child ever have to wear shoe lifts/splints?

15. How do you think he/she feels about wearing them? Different footwear/shoe modifications?

Probe: self conscious about shoe raise?

16. How does your child's leg problem affect his/her function in day-to-day activities?

17. Do you think your child worries about limping/abnormal walking/running?

18. How do you think your child accomplishes tasks compared to other kids of your age?

19. How do you feel your child's leg problems will affect his/her future function in day-to-day activities?

Experience of care

20. Is there anything you would like to comment about with regard to their hospital treatment?

21. Is there anything that could have been done better?

22. Is there anything that you wished health professionals knew or understood better about having a child with a lower limb problems?

23. Is there anything else that comes to your mind that you would like to talk about your experience of care?

Quality of life Specific Questions

24. What does Quality of Life means to you?

25. How would you describe your child's quality of life?

26. How do you think having a lower limb problem has affected your child's quality of life in general?

27. How has the lower limb problems affected your child's school?

28. How has the lower limb problems affected your child's Social life?

29. How has the lower limb problems affected your child's emotions?

30. How has the lower limb problems affected your child's behavior?

31. How has the lower limb problems affected your child's mental health?

32. How has the lower limb problems affected your child's self-esteem?

33. How has the lower limb problems affected your child's confidence?

34. How do you think we can improve the quality of life of your child?

35. How do you think your child perceives him/herself?

36. How do his/her friends react towards his/her lower limb problems?

37. How would you describe the attitudes of your extended family?

38. How would you describe the attitudes of your friends and community?

39. Are there any activities/situations you avoid because of your child's lower limb problems?

40. Is there anything else you would like to talk about your child's quality of life that we haven't covered above?

41. Can you please tell me anything that bothers you about your child's leg, even if its minor and unimportant?

Parental QOL

42. How do you think having a child with limb problem has affected your life?

43. family time

44. Emotionally

45. Social life

46. Friends

47. financially

48. Need for more support?

Are you interested in us contacting you again for cognitive interviews once we have the preliminary scale developed?
