# Supplementary material for: What matters to children with lower limb deformities: an international qualitative study guiding the development of a new patient-reported outcome measure
Source: J Patient Rep Outcomes. 2021 Apr 1;5:30. doi: 10.1186/s41687-021-00299-w (PMC8017030; doi:10.1186/s41687-021-00299-w)
Supplement: Supplementary file 2 — Additional file 2. [file 41687_2021_299_MOESM2_ESM.docx]

| **Country** | **Sex** | **Age (years)** | **Quote #** | **Child Quote ( C ), Parent Quote (P)** | **Theme** | **Sub-theme** |
| --- | --- | --- | --- | --- | --- | --- |
| India | Male | 15 | 1 | *C: I didn’t like how my leg was not straight and my knee was popping out.* | Appearance | Leg - shape |
| US | Female | 14 | 2 | *C: my one leg was longer than the other.* | Appearance | Leg - size |
| Ethiopia | Female | 17 | 3 | *C: It means by nature, my right leg was bend in ward and the left one doesn’t even touch the ground, it was short.* | Appearance | Leg - symmetry |
| Canada | Female | 11 | 4 | *P: something that really bothers me is ,because her left leg is so much shorter than her right, P: we have to sometimes get a lift in her shoe.* | Appearance | Leg - symmetry |
| US | Female | 14 | 5 | *C : Because my leg would look bent in pictures.* | Appearance | Leg - photos |
| Canada | Male | 14 | 6 | *C: I don’t like how it looks coz it just looks weird.* | Appearance | Leg - Overall |
| US | Female | 16 | 7 | *C: They would only notice how my hips were different. One was like more shaped and the other one was higher, like kind of like a balance.* | Appearance | Hip |
| India | Male | 15 | 8 | *C: I didn’t like how my leg was not straight and my knee was popping out.* | Appearance | Knee |
| Ethiopia | Male | 13 | 9 | *C: I don't like my foot because it is bent inward.* | Appearance | Foot |
| India | Female | 11 | 10 | *C: I just want them to disappear soon. They look bad.* | Appearance | Scars |
| Canada | Female | 13 | 11 | *P: She does have some quite noticeable scarring on her lower left leg. P: And in like 2 or 3 years, if there is still a lot of scarring, there is some kind of post-operative thing that they can do to kind of reduce the appearance of the scar.* | Appearance | Scars |
| Canada | Female | 8 | 12 | *C: I cannot wear short dresses and shorts in the summer.* | Appearance | Clothes - Type |
| Canada | Male | 13 | 13 | *P: Right now, he doesn’t really enjoys wearing the shorts and all the people to see the frame.* | Appearance | Clothes - Type |
| US | Male | 16 | 14 | *C: I have to wear either stretchy pants or shorts when I am in frame* | Appearance | Clothes - Type |
| Canada | Female | 11 | 15 | *P: With the frame she can only wear certain things. She knows that this is hanging out, it’s visible.* | Appearance | Clothes - Type |
| India | Female | 11 | 16 | *P: She has to either wear long pants or jeans. But now its even visible when she wears jeans.* | Appearance | Clothes - Fit |
| US | Female | 14 | 17 | *C: Shoe- lifts was probably the one thing that I probably wouldn’t go to school and , it just seemed weird cause I thought there would be inside the shoe but it wasn’t and that’s a huge thing… everybody would notice.* | Appearance | Shoe-lift |
| Canada | Female | 11 | 18 | *P: I think she wore it to school once and then she didn’t wanna wear the shoes anymore. P: it was because people were kinda pointing, you know. If it’s more visually noticeable, she’s less likely to want to do that so you know.* | Appearance | Shoe-lift |
| US | Female | 16 | 19 | *C: ...if I was to take a picture of myself standing up, I wasn’t able to do that because I didn’t feel comfortable …: like you would see me shifting. I would look like to the side and I didn’t like that because obviously people are gonna notice.* | Appearance | Pictures |
| US | Female | 16 | 20 | *C: Running? Oh, I couldn’t. It would feel like I’m—one leg is hitting the floor faster than the other, so you  would just end up like—like falling pretty much until you—unless you know how to prepare yourself. But I couldn’t run.* | Physical | Function - mobility |
| US | Female | 11 | 21 | *C: I’m slow and bad at running.* | Physical | Function - mobility |
| Canada | Male | 14 | 22 | *C: my leg problems don’t allow me to walk—like a longer period of time. After, like, about 10 – 15 minutes it will start hurting.* | Physical | Function - mobility |
| Canada | Male | 16 | 23 | *C: Well, with the frame on, obviously I feel immobile and it’s not easy or fun to not be able to do much.* | Physical | Function - mobility |
| US | Female | 8 | 24 | *C: I could not take off the clothes when I am in frames and I’m wanting to go to the mall or something, so my mom help me take them.* | Physical | Function - ADL |
| Canada | Male | 11 | 25 | *C: sometimes it was like uneven if I skated since one leg is longer than the other.* | Physical | Function - Balance |
| US | Female | 14 | 26 | *C: I stood on this leg and put this on the air then I fall over.* | Physical | Function - Balance |
| US | Female | 16 | 27 | *C: I would lose my balance and I would lean to the side.* | Physical | Function - Balance |
| India | Male | 16 | 28 | *C: I couldn’t run much when my knees used to rub against each other I. How much were you able to run I.I could run as long as I wanted to by keeping my knees apart. I had to intentionally keep them apart. I had to force my knees to stay apart while running.* | Physical | Function - Sports - Adaptation |
| Canada | Male | 14 | 29 | *C: Ah, with sports, like I said with soccer…P: … I—I play goalie…P: … so—so it’s easier for me.* | Physical | Function - Sports - Adaptation |
| India | Male | 12 | 30 | *C: When I have the frame on, I cant play.* | Physical | Function - Sports |
| US | Female | 8 | 31 | *C: Well, I don’t really like doing sports because I feel uncomfortable with my leg in the frame. C:So, I could hurt myself doing other things.* | Physical | Function - Sports |
| US | Male | 14 | 32 | *C: Well, soccer I couldn’t do. Basketball I can do cause it wasn’t as much as running as soccer.* | Physical | Function - Sports |
| US | Male | 11 | 33 | *P: because of the leg length. If he put too much pressure or if he—walked or he biked too long, his legs would hurt, he would have to stop. P: Because his legs would hurt or his hip.* | Physical | Symptoms - Pain |
| US | Female | 16 | 34 | *C: I had pain when I was in school and I was walking a lot.* | Physical | Symptoms - Pain |
| India | Male | 18 | 35 | *C: No, I can’t stand for a long time. My leg gets tired and my foot gets swollen.* | Physical | Symptoms - Tired |
| India | Male | 18 | 36 | *C: I don’t do much because of limping.* | Physical | Symptoms - Limping |
| US | Male | 16 | 37 | *C: My back always hurts and sometimes I’d have to stop doing whatever I’m doing -8CF; C: if my knee hurts, then I’ll walk slower.* | Physical | Symptoms - Pain |
| Canada | Male | 14 | 38 | *C: After, like, about 10 – 15 minutes of walking it will start hurting.* | Physical | Symptoms - Pain |
| Canada | Male | 18 | 39 | *C: ...before I got this treatment done, like it was really really bad, like I had trouble sleeping at night.* | Physical | Symptoms - Pain |
| Canada | Male | 18 | 40 | *C: Because my back has already starting to get more and more painful before I got this treatment done, like it was really bad.* | Physical | Symptoms - Pain |
| Canada | Male | 8 | 41 | *C: I don’t like how. it has the fixator on and sometimes when it's functioning it hurts, it ends up aching and pounding.* | Physical | Symptoms - Pain |
| Canada | Male | 18 | 42 | *C: It’s a little deformed, coz it’s not normal – 6CBC acquired leg deformity.* | Psychological | Body image |
| Canada | Male | 11 | 43 | *C: I’m pretty normal. I like to go to schools, which sounds kind of weird sometimes, but I like to do…sometimes I like to do sports and stuff at school. I like quality time with my family.* | Psychological | Body image |
| Canada | Male | 16 | 44 | *P: when he was going to school he wore shorts, he would wrap the shorts to fully cover: … he wanted it all wrapped up so he will get an extra T-shirt and wrap around so you couldn’t see that the bottom front of his frame and just use safety pins. he was a little bit self-conscious.* | Psychological | Body image |
| Ethiopia | Male | 16 | 45 | *C: I can find clothes but you can clearly see my leg problems when I wear shorts and I always feel ashamed wearing shorts so I don't prefer wearing them.* | Psychological | Body image |
| Canada | Female | 13 | 46 | *P: I’m really used to walking around with it and I even like—I knew how to walk without having a limp, so people didn’t even notice at all.* | Psychological | Body image |
| Ethiopia | Female | 17 | 47 | *C:I don’t go anywhere, I only go to school and church. I why? T Because I don't want to go anywhere with my stick, I just don't feel happy doing that.* | Psychological | Body image |
| Ethiopia | Male | 13 | 48 | *C: Yes, I feel so self-conscious , I look people around if they are staring at me or not.* | Psychological | Body image |
| Canada | Female | 11 | 49 | *P: she’s conscious of her appearance. She wants to be as normal, like I mentioned, if she has a lift on her leg or on her shoe, she’s very conscious about that, because you know, kids are aware of it and she is aware that they are aware of it…P: … she’s self-conscious about how she looks to the other kids.* | Psychological | Body image |
| US | Male | 16 | 50 | *C: I feel little self-conscious about the appearance of my leg with the fixator on because people may think differently of me and ask about it.* | Psychological | Body image |
| US | Female | 10 | 51 | *C: Yeah, I worry. It’s like would I still be able to move around when I grow up? Would I be able to get agile when I grow up? Will I be able to move around and agile? C: What type of job do I have to get so—with my bends legs? So, I thought about it a lot.* | Psychological | Distress - worry |
| US | Female | 9 | 52 | *C: Sometimes before I go to bed I worry about I’m never gonna be able to walk and that I might just be in the wheelchair for the rest of my life.* | Psychological | Distress - worry |
| India | Male | 14 | 53 | *C: I don’t play P.I am worried that I will hurt my foot while playing I. What do you think will happen to your foot C .It will be painful it it gets hurt while playing I: Is that why you don’t play C. Yes - 5CCH;* *P: There was a huge effect on him. He would cry watching other children, he wouldn’t be able to wear clothes.* | Psychological | Distress - worry |
| Ethiopia | Male | 16 | 54 | *C: Limping worries me a lot, some people stare at you, and kids laughs.* | Psychological | Distress - worry |
| Ethiopia | Male | 16 | 55 | *C: Yes, I worry because they told me I might not even stand by my self after a couple of years and might need a wheel chair.* | Psychological | Distress - worry |
| US | Female | 16 | 56 | *C: I always hear that like something a leg discrepancy would happen again. you never know I’m still 16. C: What if my leg decides to grow again? Then what?* | Psychological | Distress - worry |
| Ethiopia | Female | 17 | 57 | *C: I don’t go anywhere, I only went to school and church. I Because I don't want to go anywhere with my stick, I just don't feel happy doing that.* | Psychological | Distress - emotions |
| Ethiopia | Male | 13 | 58 | *C: I get mad sometimes because when I wear shorts the scar is visible and people ask me what’s wrong with me and I don't like that.* | Psychological | Distress - emotions |
| US | Female | 10 | 59 | *C:Sometimes it makes me feel sad but all that time I’m okay with it, like, when I’m not able to do things with my friends, that’s when I feel sad—that’s—why is my leg like this? I start questioning myself sometimes.* | Psychological | Distress - emotions |
| Canada | Male | 8 | 60 | *C: sometimes sadness C yeah because I keep wanting to come out of it.* | Psychological | Distress - emotions |
| Canada | Female | 11 | 61 | *C: Like, sometimes this thing (frame) makes me really angry [chuckle]… P: … and then I become mad at the world and just everything.* | Psychological | Distress - emotions |
| India | Female | 11 | 62 | *C : Yes, I do get angry because I want it to get better quickly.* | Psychological | Distress - emotions |
| Canada | Female | 13 | 63 | *C: In the beginning, ah, like, before I had it, I was like so mad, like, I was like wondering why I had to have this. None of my friends had anything like it so I felt like really alone and stuffs.* | Psychological | Distress - emotions |
| US | Female | 16 | 64 | *C: I would get kind of upset and bring myself down.* | Psychological | Distress - emotions |
| India | Male | 18 | 65 | *C: Yes, I feel angry I. Who do you get angry at C. At my leg that limps.* | Psychological | Distress - emotions |
| India | Male | 14 | 66 | *C: Yes, I would get angry when I was not able to play.* | Psychological | Distress - emotions |
| US | Female | 16 | 67 | *M: she was concerned about, “Mommy how is—how it’s gonna be after surgery, how …” She was thinking about how—how she’s gonna look …P:… after the surgery.* | Psychological | Distress - emotions |
| US | Male | 14 | 68 | *C: I felt a little bit annoyed because, like, it was kind of normal as, like, people are, like, always asking. So, it’s just, like, when can you stop asking?* | Psychological | Distress - emotions |
| US | Female | 16 | 69 | *C: I didn’t even wanna get dressed in the mornings. Like I didn’t even wanna get ready for anything anymore. I wanted to wear like baggy clothes. P: So it did affect my confidence somewhat and my self-esteem.* | Psychological | Confidence and Self-esteem |
| Ethiopia | Male | 16 | 70 | *C: Always get very sad, seeing every kid play and not playing. And ask question why this happened to me and feel inferior than the other kids.* | Psychological | Confidence and Self-esteem |
| US | Male | 16 | 71 | *C: Well, right now with the frame on, I feel, like, not very confident in my abilities because I really can’t do as much because I don’t have my full range of motions.* | Psychological | Confidence and Self-esteem |
| Canada | Female | 11 | 72 | *P: when the frame is on, her self-confidence kind of gets a little less.. Just because she is aware of it and she knows \that they’re looking at her.…P… —I know she doesn’t like that as well. She doesn’t like to draw attention to it.* | Psychological | Confidence and Self-esteem |
| US | Female | 10 | 73 | *C: And I’m not confident to do a lot of things most of the time I will ch—like if it’s a big events I’m not really confident to go with something short. I try to find something long to cover my legs …C: So, it reduces my confidence.* | Psychological | Confidence and Self-esteem |
| US | Female | 9 | 74 | *C: Yes, my confidence is affected. I: Yes. In—in what way do you …C: Usually when I go to school wearing—wearing shorts, skirt cause when my leg shows I usually wanna cover it, like wrap a thing around it, so nobody can see it.* | Psychological | Confidence and Self-esteem |
| US | Female | 16 | 75 | *C: if I was to take a picture of you standing up, I wasn’t able to do that because I didn’t feel comfortable .like you would see me shifting. I would look like to the side and I didn’t like that because obviously people are gonna notice. So I couldn’t even do that. I never like—like to be taken pictures of like full body and then I would l look at myself and be like, “Wow, like this is tremendous, this is—“ So imagine somebody that has a bigger difference than me :… that’s even worse.* | Psychological | Confidence and Self-esteem |
| US | Female | 8 | 76 | *C: feel better—I bought a—a journal so I can write about it sometimes.* | Psychological | Coping |
| US | Male | 16 | 77 | *C: Sometimes I would be sad because it’s like—it’s like, “Why me?” but then it’s, like, everything happens for a reason.* | Psychological | Coping |
| Canada | Male | 14 | 78 | *C: Ah, I just think that there are some people who are far worst…P: … who have no legs. I: So that makes you feel better then, right? C: Well it makes my life—makes me feel like my problems are not as bad.* | Psychological | Coping |
| US | Male | 16 | 79 | *C: It’s a reminder of what I have been through. C:How far I’ve come. C:And just a part of me and sometimes people notice them and they ask me what happened.* | Psychological | Coping |
| US | Male | 14 | 80 | *C: but sometimes I think like, it is better because I get experience from this and I get to grow from this, like …:… like I overcame, like, this whole problem …C:… by myself …C:… and I’m proud of it.* | Psychological | Coping |
| US | Female | 16 | 81 | *C:I also didn’t want that to affect me and what I wanted to be. I wasn’t gonna let it take over me.* | Psychological | Coping |
| US | Female | 14 | 82 | *C:Usually if you find out that something’s wrong with you, it kinda push you down but it actually brought me up. It made me wanna do things better.* | Psychological | Coping |
| Canada | Male | 11 | 83 | *C: But we do have appointments for like physio and going to the doctor’s appointment – that would kind of affect my school because on school days, I would have to go to the hospital sometimes, so I would have to minimize my going to school, and then I’d have to work a bit harder, because of the days that I miss school…I have to get the homework for that.* | School | Function |
| Ethiopia | Male | 11 | 84 | *C: No I don't go to school when I have the frame, I’m scared kids might push me and if I fell there will be a lot of problem.*. | School | Function |
| US | Female | 14 | 85 | *C: I couldn’t go to school if I’m on medication. So that’s why it took me so long to go to school because I don’t wanna go to school in pain and I couldn’t take Advil.* | School | Function |
| Canada | Male | 11 | 86 | *C: It’s bit nervous going to school with the frame because I’m a bit nervous because there’s a lot of kids at school and a lot of curious kids that might want to like touch it and I don’t want them to touch it in case of infection.* | School | Function |
| Ethiopia | Male | 10 | 87 | *C: When I was sick we went to different hospitals and miss a lot of school and I forgot most of it so I start from grade one.* | School | Function |
| Ethiopia | Female | 11 | 88 | *C: Ohh I stopped school for a year and I was not able to play with my friends either.* | School | Function |
| Ethiopia | Female | 17 | 89 | *C: Yes a lot, for my first surgery I stayed in the hospital for a couple of weeks and for the second one I stayed here for a couple of months.* | School | Function |
| Ethiopia | Male | 11 | 90 | *C: I had to quit school and that was hard because it takes a lot of time to get the treatment.* | School | Function |
| India | Male | 15 | 91 | *C: This has affected my studies a lot. My whole school year has been wasted.* | School | Function |
| Ethiopia | Male | 11 | 92 | *C: I could not be equal to my friends, not being able to be in the grade i want to be in my age.* | School | Function |
| India | Male | 12 | 93 | *C: I don’t play at school. I am worried that I will hurt my foot while playing.* | School | Participation |
| Canada | Male | 14 | 94 | *C: I—when we do a running thing… P: … ah, ah, usually I just sit out coz as I got older when I started to run it got more painful and harder, so now I just kinda—when they are doing, like, a running activity I usually just don’t do it coz it hurts.* | School | Participation |
| India | Male | 12 | 95 | *C: No, I don’t play outdoors when I have the frame. I play indoor game that I can play sitting down. My friends also come and play with me.* | School | Participation |
| US | Female | 8 | 96 | *C: Well, when I wasn’t re—ready to keep-up with the class, the teacher sent me a kid to walk with me.* | School | Emotional support |
| US | Female | 12 | 97 | *C:Um, they were really good. They helped me if I needed it. C: They helped against bullies and stuff like that.* | School | Emotional support |
| India | Male | 12 | 98 | *C: Yes, I have a lot of friends. In my last school, the entire school became my friend because they all would come to see me after my operation.* | School | Emotional support |
| Ethiopia | Female | 17 | 99 | *C: When I go to school I used a stick to walk and had to take a stair to get to class and my hands always get bloody and I can’t carry my text books I have to ask my friends to carry them for me.* | School | Instrumental Support |
| Ethiopia | Female | 11 | 100 | *C: They are supportive. In our break or after school they always let me out first or stay with me until the crowed is gone so that I won’t fall and hurt myself again.* | School | Instrumental Support |
| Canada | Male | 16 | 101 | *C: Usually teachers just make sure someone brings my backpack to me and overall it’s fine so far.* | School | Instrumental Support |
| Ethiopia | Female | 17 | 102 | *C: And if there is a makeup class the teachers always give that in the top of the building, because all of the classes will be occupied and I can’t take all of the stairs so I had to miss all of the makeup classes.* | School | Environmental Barriers |
| US | Female | 16 | 103 | *C: It made it so much harder to keep up with all the work and just like, even now, I feel so overwhelmed because I’m in a wheelchair, I don’t have a desk, like my papers are everywhere. I ha—where am I gonna write.* | School | Environmental Barriers |
| US | Female | 16 | 104 | *C: I have to deal with whatever—whatever I have. So in the negative aspect as people, like, kids my age these days, like I’m in 11th grade, I’m in high school, people look at me like, “What is wrong with you?”* | School | Isolation |
| US | Female | 14 | 105 | *C: Some people were really mean to me. Some people would start out dress code to me because I physically could not put on pants. Because the brace came up to here.* | School | Isolation |
| Canada | Female | 8 | 106 | *C: It’s an ongoing problem with the kids at school who always ask me “what do you have in your feet”. I don’t like that.* | School | Isolation |
| Canada | Female | 13 | 107 | *C: Oh ah [pause]—I don’t—well, yeah, coz sometimes we went on field trips and stuff and I was kind of stuck in my crutches watching everyone. I was kinda left out sometimes… coz everyone was riding around and I was just sitting there.* | School | Isolation |
| Canada | Male | 11 | 108 | *C: But sometimes people would be curious about my leg problems, and sometimes they’d wonder, ‘Oh why is your shoe bigger than the other shoe?’* | School | Isolation |
| US | Male | 16 | 109 | *I: Did you ever hesitate to make more friends or new friends because you were worried that they were going to notice your leg? C:Occasionally, I might, cause I think before like, analyzing people to see how they may react to it and if they were good person and they will look back, well I would be more open to them but as opposed to a person who might not be as supportive.* | Social | Function |
| US | Female | 16 | 110 | *C: But the moment when I would walk again, you could notice. That made me feel very like uncomfortable. C:So I never wanted to go out. If my mom told me, “Oh, let’s go somewhere and let’s go to the grocery store.” Anywhere, like I didn’t wanna go. I just wanted to stay home in comfortable clothes where nobody would know what I had.* | Social | Function |
| US | Female | 16 | 111 | *C: I wouldn’t wanna go out with friends because they were normal. My friends were normal.They look even and I didn’t. And I always like thought and I was to the side.* | Social | Function |
| US | Female | 16 | 112 | *C: I’m in high school—I would like be on the social media and see everybody else taking picture of like, their body and stuff like, you know somebody else I guess.* | Social | Function |
| US | Male | 16 | 113 | *C: Sometimes I’ll be sad because I—I wasn’t able to go to the beach with my friends or go to amusement parks* | Social | Function |
| India | Male | 12 | 114 | *C: Can’t play catch with my siblings. I. What do you do when they are playing catch C. sit on one side* | Social | Function |
| Canada | Male | 11 | 115 | *P: A little bit inconvenient right now for me because he has to almost all the time stay at home, not going out with us – well, we didn’t going out on weekends. We cannot have a long trip for a while, because we have to take care of the alignment, cleaning of pins, those kind of things – so after the surgery, we stay home more than before.* | Social | Function |
| Canada | Male | 18 | 116 | *C: It’s harder to get a job. It’s been a couple of months and I’m looking and even places that are hiring, uh, they don’t call back.* | Social | Function |
| Canada | Male | 18 | 117 | *C: I worked in sales. It’s all standing-up jobs. Ah um, I would have done apprenticing if it weren’t for the high demand of electrical apprentice, if it weren’t for the amount of standing that I had to do…* | Social | Function |
| India | Male | 12 | 118 | *C: No, My other friends tell the new friends not to ask me about it because I might feel bad about it.* | Social | Emotional support |
| Canada | Female | 13 | 119 | *C: They're really, really helpful, especially when I had my frame on. P:Um, they would carry my bags, they would carry my instrument, and they didn’t care that I was, like—coz I was in a wheelchair. So it’s like, when I had to out places— C:So then, they even took me to concerts in my wheelchair and then they took me trick or treating.* | Social | Emotional support |
| US | Female | 16 | 120 | *I: Do you—do you—so you did feel the need of support groups? P: Yeah. Of course and they need … to have people that understand what they went through …P:… or what they’re going through.* | Social | Emotional support |
| Ethiopia | Female | 17 | 121 | *C: My friends are so understanding, starting from grade one until grade nine they are the one who carries my textbooks. And I never went to school alone, they always come in turn to my home and go to school with me.* | Social | Emotional support |
| Canada | Male | 16 | 122 | *C: A lot of strangers open doors for me when I had the frame on, then people would open a door if they saw me with my crutches.* | Social | Emotional support |
| US | Female | 14 | 123 | *C: My mom was the only person that was helpful. She was so supportive. Um, I definitely think it—it was stressful. She had to take a lot of time off of work to take me and …P:… pick me up.* | Social | Emotional support |
| Canada | Female | 13 | 124 | *C: They're really, really helpful, especially when I had my frame on. P: Um, they would carry my bags, they would carry my instrument, and they didn’t care that I was, like—coz I was in a wheelchair. So it’s like, when I had to out places— P:So then, they even took me to concerts in my wheelchair and then they took me trick or treating.* | Social | Instrumental Support |
| India | Male | 14 | 125 | *C: Yes, everyone knew and even though some would tease me, the rest were okay with it.* | Social | Isolation |
| India | Male | 18 | 126 | *C: Yes, they do make fun of me. They call me lame .* | Social | Isolation |
| US | Female | 10 | 127 | *P: She would go to church, she would like to sit inside the church …F:… because people were looking at her F:She would like to want that she would sit outside.* | Social | Isolation |
| Canada | Male | 13 | 128 | *C: I get upset and like leave when my siblings make fun of me.* | Social | Isolation |
| Ethiopia | Female | 11 | 129 | *I: What happens when someone new meets you for the first time? C: They pity me and always has a sad face . B What does that make you feel? C: It makes me very mad.* | Social | Isolation |
| US | Male | 16 | 130 | *C: They would go call me cyborg.* | Social | Isolation |
| Canada | Male | 8 | 131 | *P: yeah, and just when we're in places he's really looking at your eyes, he pays attention to people staring at him.* | Social | Isolation |
| Canada | Female | 13 | 132 | *C: In the beginning, ah, like, before I had it, I was like so mad, like, I was like wondering why I had to have this. None of my friends had anything like it so I felt like really alone and stuffs.* | Social | Isolation |
| Canada | Female | 11 | 133 | *C: Um, kinda just because they look at me differently than other people  And, um, they don’t wanna social, like, talk and stuff.* | Social | Isolation |
| Canada | Female | 13 | 134 | *C:.. So I just started showing my leg when we went somewhere and I just kind of find it funny how people would stare at me and like they would make like really funny faces when they looked at my leg.* | Social | Isolation |
| India | Female | 12 | 135 | *C: Yes, my friends would notice and ask what happened. C: They would say there is a bit of a limp.* | Social | Isolation |
| US | Male | 14 | 136 | *P: because at this point, you know, mostly everybody that he came close to will have something to say about him …P:… and about his leg and … about the way he looked.* | Social | Isolation |
